# Tincture of Iodine in Bubo

**Published:** 1855-11

**Authors:** 


Tincture of Iodine in Bubo.—Mr. Joliclerc states as the result of much observation, that tincture of iodine is the best application in bubo, preventing or dissipating fluctuation, and facilitating healing if the bubo be already open. He applies it by gentle friction, so as to avoid vesication.—(*Ibid.*)

